# Interleukin-10 regulates the inflammasome-driven augmentation of inflammatory arthritis and joint destruction

**DOI:** 10.1186/s13075-014-0419-y

**Published:** 2014-08-30

**Authors:** Claire J Greenhill, Gareth W Jones, Mari A Nowell, Zarabeth Newton, Ann K Harvey, Abdul N Moideen, Fraser L Collins, Anja C Bloom, Rebecca C Coll, Avril AB Robertson, Matthew A Cooper, Marcela Rosas, Philip R Taylor, Luke A O’Neill, Ian R Humphreys, Anwen S Williams, Simon A Jones

**Affiliations:** Cardiff Institute of Infection and Immunity, The School of Medicine, Cardiff University, Heath Park Campus, Cardiff, CF14 4XN Wales UK; Trinity Biomedical Sciences Institute, School of Biochemistry and Immunology, Trinity College Dublin, 152-160 Pearse Street, Dublin 2, Ireland; Institute for Molecular Bioscience, The University of Queensland, St Lucia Campus, Brisbane 4072, QLD Australia

## Abstract

**Introduction:**

Activation of the inflammasome has been implicated in the pathology of various autoinflammatory and autoimmune diseases. While the NLRP3 inflammasome has been linked to arthritis progression, little is known about its synovial regulation or contribution to joint histopathology. Regulators of inflammation activation, such as interleukin (IL)-10, may have the potential to limit the inflammasome-driven arthritic disease course and associated structural damage. Hence, we used IL-10-deficient (IL-10KO) mice to assess NLRP3 inflammasome-driven arthritic pathology.

**Methods:**

Antigen-induced arthritis (AIA) was established in IL-10KO mice and wild-type controls. Using histological and radiographic approaches together with quantitative real-time PCR of synovial mRNA studies, we explored the regulation of inflammasome components. These were combined with selective blocking agents and *ex vivo* investigative studies in osteoclast differentiation assays.

**Results:**

In AIA, IL-10KO mice display severe disease with increased histological and radiographic joint scores. Here, focal bone erosions were associated with increased tartrate-resistant acid phosphatase (TRAP)-positive cells and a localized expression of IL-1β. When compared to controls, IL-10KO synovium showed increased expression of *Il1b*, *Il33* and NLRP3 inflammasome components. Synovial *Nlrp3* and *Casp1* expression further correlated with *Acp5* (encoding TRAP), while neutralization of IL-10 receptor signaling in control mice caused increased expression of *Nlrp3* and *Casp1*. In *ex vivo* osteoclast differentiation assays, addition of exogenous IL-10 or selective blockade of the NLRP3 inflammasome inhibited osteoclastogenesis.

**Conclusions:**

These data provide a link between IL-10, synovial regulation of the NLRP3 inflammasome and the degree of bone erosions observed in inflammatory arthritis.

**Electronic supplementary material:**

The online version of this article (doi:10.1186/s13075-014-0419-y) contains supplementary material, which is available to authorized users.

## Introduction

As new biologics enter the clinical arena and advances in synovial histopathology identify divergent mechanisms of arthritis progression, it is essential to understand how the cytokine network governs the pattern of synovial inflammation [1,2]. Innate sensing mechanisms involving pattern recognition receptors are increasingly implicated in autoimmunity and promote cytokine responses associated with rheumatoid arthritis. Although these pathways represent promising therapeutic targets, further investigation is required to understand the expression and functional contribution of pattern recognition receptors in autoimmunity.

Pattern recognition receptors were initially characterized as sensors of microbial products of bacterial, fungal or viral infection. These include the Toll-like receptors (TLR); nucleotide-binding domain and leucine-rich repeat containing receptors (NLR), Rig-I-like receptors (RLR) and C-type lectins [3,4]. Activation of these receptors promotes the inflammatory regulation of various interleukins, tumour necrosis family members and type-1 interferons [3]. They therefore represent innate sensing mechanisms, which shape the adaptive immune response to chronic disease, allergy, cancer and infection. As a consequence, various processes have evolved to protect against the prolonged activation of these receptors. For example, interleukin (IL)-10 limits the duration and intensity of their signaling in myeloid cells [5,6]. Here, IL-10 inhibits pattern recognition receptor signaling through mechanisms, which include downregulation of MyD88 expression [7], and the ubiquitination and subsequent degradation of MyD88-dependent signaling molecules such as TRAF6 [8]. In experimental models of inflammatory arthritis, IL-10 is protective and mice deficient in IL-10 show exacerbated joint inflammation [9,10]. These data are consistent with the characterization of IL-10 as a cytokine synthesis inhibitory factor, which acts as an immunomodulatory cytokine affecting both innate and cellular immunity [11,12]. For example, IL-10 inhibits nuclear factor kappa B (NF-κB) signaling in response to TLR agonists to block expression of certain proinflammatory mediators associated with arthritis progression. Interleukin-10 is abundantly expressed in synovial fluids of RA patients and has been linked with the control of bone resorption through inhibition of osteoclastogenesis *in vitro* [13-15].

While IL-10 is renowned for its ability to inhibit TLR signaling, its impact on innate sensing receptors, including the NLR family, is less documented. Here, caspase 1 activity is essential for the processing of cytokine precursors (for example, pro-IL-1β, pro-IL-18 and potentially pro-IL-33) into functionally active mature forms [16]. Activation of caspase 1 by the NLR family member NLRP3, acting in association with its adaptor protein ASC, leads to the secretion of IL-1β and IL-18 [16]. Each of these cytokines affect arthritis progression [17,18], which suggests that innate sensing complexes (termed the inflammasome) must be regulated during the course of disease. Various particulate and crystalline agonists activate the NLRP3 inflammasome. For example, monosodium urate crystals and calcium pyrophosphate dihydrate crystals trigger arthritis symptoms in inflammatory forms of gout and pseudogout [19-21], while basic calcium phosphate (hydroxyapatite) crystals are seen in 70% of osteoarthritis (OA) cases [22,23]. A role for the NLRP3 inflammasome in arthritic disease is illustrated by several *in vitro* studies, which show that crystals from the joints of OA patients and basic calcium phosphate crystals induce IL-1β production by macrophages [24,25]. Also, a recent report shows that there is modulation of the NLRP3 inflammasome in peripheral blood mononuclear cells in RA patients and that single nucleotide polymorphisms (SNPs) in *NLRP3* are associated with disease severity [26]. However, little is known about the regulation and activation of inflammasome components in inflammatory arthritis. We now show that the exacerbated joint pathology seen in IL-10KO mice during antigen-induced arthritis (AIA) is associated with increased synovial expression of NLRP3 inflammasome components and a localized expression of IL-1β at sites of focal bone erosions. Our data supports a role for IL-10 as a negative regulator of the inflammasome and highlights a role for the inflammasome in osteoclastogenesis during inflammatory arthritis.

## Materials and methods

### Mouse strains

Inbred C57BL/6 mouse strains from The Jackson Laboratory (Bar Harbor, ME, USA) were bred and maintained in-house under high barrier and pathogen-free conditions. All animal studies were performed in the United Kingdom. Experiments were performed on eight- to twelve-week-old male mice in accordance with UK Home Office Project License PPL-30/2361 and 30/2928. The ethical approval of these licenses covers all aspects of the study and all experiments conducted.

### Induction of murine AIA

Mice were immunized (subcutaneous (s.c.)) with an emulsion containing 1 mg/ml methylated bovine serum albumin (mBSA) in phosphate-buffered saline (PBS) and Freud’s complete adjuvant (CFA) (Sigma-Aldrich, St Louis, MO, USA). Concurrently, mice were injected (intraperitoneal (i.p.)) with 200 ng of heat-inactivated *Bordetella pertussis* toxin adjuvant (Sigma-Aldrich, Poole, UK). The immune response was boosted one week later with a second injection (s.c.) of mBSA emulsified in CFA. Arthritis was induced two weeks later with an intra-articular (i.a.) injection of 10 μl of mBSA (10 mg/ml) into the right knee joint. Arthritis progression was monitored using a micrometer to measure changes in knee joint swelling.

### Radiology

A Kodak *in vivo* Imaging System FX was used to take radiographs of the mouse knee joints. Both arthritic (right) and non-arthritic joints (left) were compared. Radiographic scores were independently assigned by an orthopedic registrar and based on visible bone erosions (0; normal, 1; mild, 2; moderate, 3; severe).

### Histology

Joints were fixed in neutral-buffered formalin saline, decalcified with formic acid at 4°C and embedded in paraffin. Midsagittal sections (8 μm) were stained with haematoxylin, safranin-O and Fast Green. Two independent observers scored histology sections for subsynovial inflammation (0 = normal, to 5 = ablation of adipose tissue due to leukocyte infiltrate), synovial exudate (0 = normal, to 3 = substantial number of cells with large fibrin deposits), synovial hyperplasia (0 = normal 1 to 3 cells thick, to 3 = over three layers thick with overgrowth onto joint surfaces with evidence of cartilage/bone erosion), cartilage/bone erosion (0 = normal, 3 = destruction of a significant part of the bone). Cartilage integrity was determined in histological sections using a Mankin scoring system. Two independent observers evaluated cartilage irregularity and cleft formation (0 = normal, to 6 = complete disorganization of glycoproteins with clefts into the cartilage), cellularity (0 = normal, to 3 = hypocellularity), proteoglycan depletion (0 = normal, to 4 = complete proteoglycan degradation with no dye apparent) and tidemark integrity (0 = intact, to 1 = tidemark crossed by blood vessels). The total sum of these scores resulted in a maximum score of 14. For detection of tartrate-resistant acid phosphatase (TRAP) activity, slides were rehydrated after decalcification, incubated with TRAP staining solution (0.2 M acetate buffer, 50 mM sodium tartrate, 0.5 mg/ml naphthol AS-MX phosphate, 1.1 mg/ml Fast Red Violet LB salt) and counterstained with haematoxylin.

### Immunohistochemistry

Antigen retrieval was performed on paraffin-embedded sections using either trypsin (0.1%) for 30 mins at 37°C, or 10 mM citrate buffer (pH 6) for 40 mins at 95°C. Endogenous peroxidase and biotin activity was blocked using 3% H_2_0_2_ and an avidin/biotin blocking kit (Vector Laboratories, Burlingame, CA, USA) respectively. Sections were incubated in 10% (v:v) rabbit serum for 1 hour before staining with rat anti-mouse F4/80 (1:50 dilution, Santa Cruz Technology, Santa Cruz, CA, USA). Antibody binding to sections was detected with rabbit anti-rat biotin-conjugated secondary antibody and streptavidin-horseradish peroxidase (HRP) complex (Vector Laboratories). Diaminobenzidine substrate (Dako, Glostrup, Denmark) was used to develop sections and haematoxylin was used as a counterstain.

### Image analysis

Immunohistochemistry was viewed with a Leica DMLB light microscope (Milton Keynes, UK). Analysis across five random fields of view was performed using the Leica digital image capture program. Values are expressed as a percentage of total immunoperoxidase staining.

### Osteoclast cell culture and TRAP stain

Bone marrow cells from femurs of WT and IL-10KO mice were re-suspended in alpha minimum essential medium (αMEM) supplemented with 10% (v:v) foetal calf serum (FCS) and seeded at a density of 6.4 × 10^6^ cells/ml in 24-well plates. Following adhesion culture media was supplemented with macrophage colony-stimulating factor (MCSF) and receptor activator of NF-κβ ligand (RANKL). IL-10 and inflammasome inhibitors were added as indicated in figure legends. TRAP-positive cells were detected after seven days and total RNA isolated from subsequent analysis.

### Quantitative real-time PCR (qPCR)

Synovial membranes were dissected from the underpinning cartilage of knee joints [27]. Total RNA was extracted from samples using TRI Reagent (Sigma-Aldrich) and cDNA derived from 1 μg of total RNA using a reverse transcription kit (Primer Design, Southampton, UK) [27]. Gene expression analyses were performed on triplicate samples with SYBR Green (Invitrogen, Thermo Fisher Scientific, Carlsbad, CA, USA) using an ABI Prism 7900HT instrument (Applied Biosystems, Thermo Fisher Scientific, Carlsbad, CA, USA). Details of oligonucleotide primer sequences are presented in Additional file 1. Data analysis was performed using the Sequence Detection System Version 2.3 software (Applied Biosystems).

### Enzyme-linked immunosorbent assay (ELISA)

Serum dickkopf-1 (DKK1) was quantified using a commercial enzyme-linked immunosorbent assay (ELISA) (R&D Systems Inc, Minneapolis, MN, USA) as per the manufacturer’s instructions.

### Statistical analysis

Data was evaluated using the non-parametric Mann-Whitney *U* test and an unpaired Student *t* test. In all cases, *P* <0.05 was considered significant.

## Results

### IL-10KO mice display increased disease severity in experimental arthritis

**Figure 1:**
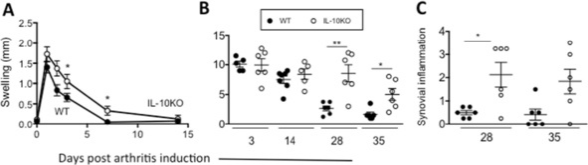
**IL-10KO mice have enhanced and prolonged antigen-induced arthritis (AIA).** Disease activity in AIA challenged WT (closed circles) and IL-10KO (open circles) mice. **(A)** Joint swelling, **(B)** arthritic index scores and **(C)** synovial inflammation are shown. Swelling data reflects the mean ± SEM of knee diameters (in mm; n = 7-28, ^*^ *P* <0.05). Arthritic index and synovial inflammation values post AIA are presented for individual joints taken at days 3, 14, 28 and 35 (data is presented as the mean ± SEM, n = 5-7 per time point from a single experiment, ^*^ *P* <0.05, ^**^ *P* <0.01 between WT and IL-10KO groups). IL-10KO, interleukin-10-deficient; WT, wild-type.

In line with an anti-inflammatory role for IL-10 in experimental arthritis, AIA in IL-10KO mice caused increased joint swelling and an overall exacerbation of joint pathology as compared to wild-type (WT) controls (Figure 1A and B). Evaluation of synovial inflammation based on synovitis, cellular infiltrate, exudate and joint damage showed that histopathology was more prolonged in IL-10KO mice (Figure 1C). Thus, IL-10 acts to prevent long-term subclinical disease and the promotion of self-limiting synovitis.

### IL-10-deficiency is associated with loss of cartilage and increased bone erosion

**Figure 2:**
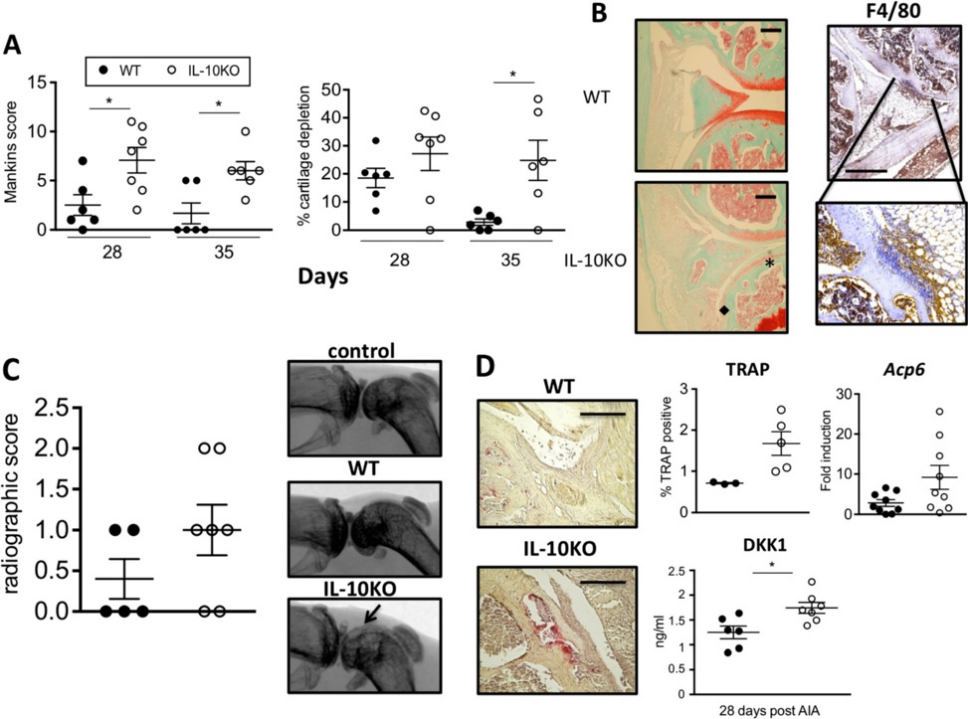
**Bone erosions are augmented in the IL-10KO joints following antigen-induced arthritis (AIA). (A)** Mankin’s score (indicating severity of cartilage damage) (left) and percentage of cartilage degradation of mouse joints (right) at day 28 and 35 post arthritis induction (mean ± SEM, n = 6-7 mice/time point from a single experiment; *P* <0.05 between WT (closed circles) and IL-10KO (open circles) groups). **(B)** Representative Haematoxylin and ‘safranin-O Green-stained parasagittal joint sections (left) taken on day 35 are shown for WT and IL-10KO mice. The star indicates cartilage degradation and diamond shows bone erosion (scale bar: 100 μm). Representative F4/80 staining (right) adjacent to bone erosions in joint sections (scale bar: 500 μm) from IL-10KO mice three days post AIA (right). **(C)** Radiographic scoring (left) and representative radiographic images (right) of the joints of WT and IL-10KO mice 28 days post arthritis induction. Representative images are presented from a single experiment using six to eight mice per mouse strain. **(D)** WT and IL-10KO joints were stained for TRAP positivity (left) on day 28 post AIA (scale bar: 200 μM) and quantified by computer analysis (top middle) (n = 3-5 mice), and expression in the joints of osteoclast marker; *Acp6* was determined by qPCR and made relative to the values for *18S* ribosomal RNA (n = 9 mice) (top right). Serum DKK1 (bottom) was detected using commercial ELISA in samples obtained from challenged WT and IL-10KO mice (n = 6-7 mice/mouse strain, ^*^ *P* <0.05). DKK1, dickkopf-1; ELISA, enzyme-linked immunosorbent assay; IL-10KO, interleukin-10-deficient; qPCR, quantitative real-time PCR; TRAP, tartrate-resistant alkaline phosphatase; WT, wild-type.

To determine the impact of joint inflammation on degenerative processes within the inflamed synovium, cartilage damage was based on a Mankin’s score and quantified as a percentage loss of safranin-O staining (Figure 2A). When compared to WT mice, cartilage depletion was more pronounced in IL-10KO mice at day 28 and 35 post arthritis induction (Figure 2A). Histology sections from IL-10KO mice also showed evidence of increased bone damage and sites of focal bone erosions. While no significant difference in the quantification of synovial F4/80 staining was observed in synovial tissue for WT and IL-10KO mice, an increased localization of F4/80 staining was observed around sites of focal bone erosions in sections from AIA-challenged IL-10KO mice (Figure 2B). Radiographic assessments of arthritic joints supported this finding and revealed a more pronounced loss of bone integrity in IL-10KO mice (Figure 2C). To substantiate an involvement of IL-10 in bone turnover, the presence of TRAP-positive osteoclasts was detected (Figure 2D). Quantification of TRAP stain showed an increase in TRAP-positive cells in joint sections from IL-10KO mice (Figure 2D). In accordance, elevated *Acp5* (encoding TRAP) gene expression was also observed in synovial mRNA from IL-10KO mice (Figure 2D). This was mirrored by a systemic increase in the surrogate bone erosion marker, DKK-1 (Figure 2D).

### Focal bone erosions are associated with localized IL-1β production

**Figure 3:**
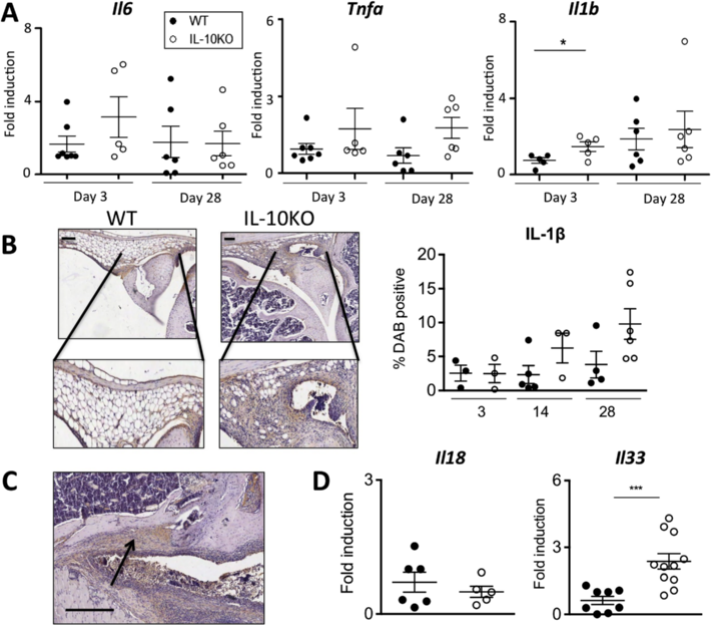
**Augmented IL-1β in the IL-10KO mice following antigen-induced arthritis (AIA). (A)** Expression of inflammatory cytokines; *Il6, Tnf* and *Il1b* were examined in the synovial mRNA of WT (closed circles) and IL-10KO (open circles) mice 3 and 28 days after AIA induction and made relative to the values for *18S* ribosomal RNA (n = 4-7, ^*^ *P* <0.05). **(B)** Representative immunohistochemistry (left) and computer-quantified scoring (right) of IL-1β staining in joint sections (scale bar: 100 μm) for WT and IL-10KO joints. Results show individual joints taken over the course of disease in AIA-challenged mice (n = 3-6). **(C)** Representative immunohistochemical staining for areas of focal IL-1β secretion in IL-10KO bone 28 days post AIA (scale bar: 300 μm). **(D)** *Il18* and *Il33* were examined in synovial mRNA from WT and IL-10KO mice taken three days after AIA induction. Values were made relative to *18S* ribosomal RNA (n = 5-11, ^***^ *P* <0.001). IL-10KO, interleukin-10-deficient; WT, wild-type.

To define the mechanisms governing bone damage in IL-10KO mice, we next considered the inflammatory environment of the inflamed synovium. Total mRNA was extracted from the synovium of AIA-challenged mice (taken at day 3 post AIA to reflect the initiation of disease) and cytokine gene expression compared in IL-10KO and WT mice. While IL-10KO mice showed no substantive alteration in synovial *Il6* or *Tnf* over 28 days, there was a twofold increase in *Il1b* over that seen in WT mice three days after arthritis induction, which had resolved by day 28 (Figure 3A). Increased synovial IL-1β expression was also observed by immunohistochemistry (Figure 3B). Interleukin-1β protein staining was most prominent in joint sections from IL-10KO mice at day 28 post AIA and coincided with the observed elevation in bone erosion. In this regard, IL-1β staining co-localized to areas of focal bone erosion (Figure 3C). Other IL-1 family members, including IL-18 and IL-33 have also been linked with bone turnover [28,29]. Synovial mRNA from AIA-challenged IL-10KO mice showed no change in *Il18* expression, but a 3.5-fold increase in *Il33* as compared to WT controls (Figure 3D). These data highlight a specific role of IL-1 family members in the destructive processes of inflammatory arthritis.

### IL-10 regulates inflammasome expression within the inflamed synovium

**Figure 4:**
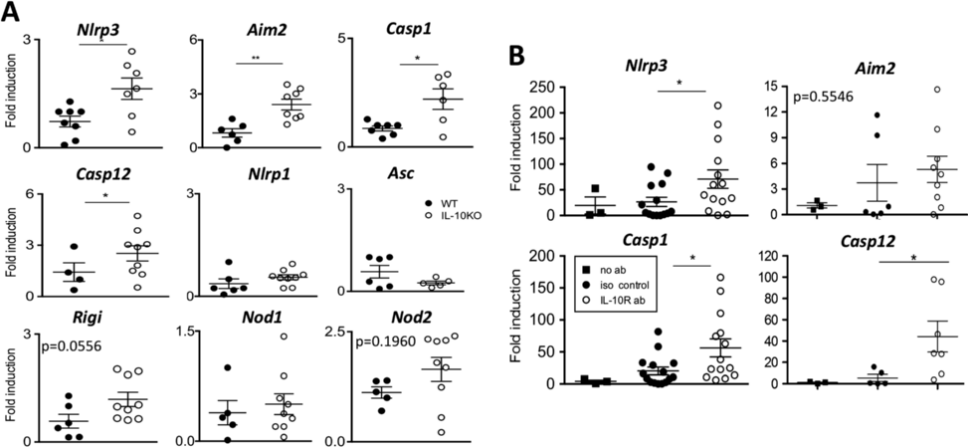
**IL-10 modulates specific inflammasome component expression during antigen-induced arthritis (AIA). (A)** Expression of inflammasome components; *Nlrp3, Aim2, Casp1, Casp12, Nlrp1, Asc, Rigi, Nod1* and *Nod2* were examined in synovial mRNA of WT (closed circles) and IL-10KO (open circles) mice three days after AIA induction. Values are relative to *18S* ribosomal RNA (n = 5-9, ^*^ *P* <0.05). **(B)** Changes in *Nlrp3, Aim2, Casp1* and *Casp12* in WT joint sections treated without/with intra-articular-administrated anti-IL-10R or isotype control antibody at the onset of AIA. Values are presented for individual joints taken at day 3 post AIA and are relative to *18S* ribosomal RNA (n = 3-15, ^*^ *P* <0.05). IL-10KO, interleukin-10-deficient; WT, wild-type.

Activation of the inflammasome is required for the generation of active IL-1β. We therefore examined the synovial regulation of inflammasome components by IL-10 during arthritis. At day 3 post AIA induction, synovial *Nlrp3*, *Aim2*, *Casp1* and *Casp12* were all significantly elevated (approximately two- to threefold) in IL-10KO mice when compared to WT controls (Figure 4A). In contrast, *Nlrp1*, *Asc*, *Rigi*, *Nod1* and *Nod2* remained largely unaltered from WT mice (Figure 4A). To verify a link between IL-10 and synovial inflammasome expression, WT mice were administered (i.a.) with a blocking anti-IL-10R antibody at the onset of AIA (Figure 4B). When compared to isotype or vehicle-control treatments, inhibition of IL-10R signaling promoted a three- to fourfold increase in synovial *Nlrp3, Casp1* and *Casp12,* while *Aim2* showed a trend towards significance (Figure 4B). Thus, confirming a link between IL-10 and regulation of the *Nlrp3* inflammasome within the inflamed synovium.

### Regulation of osteoclastogenesis by IL-10 and the inflammasome

**Figure 5:**
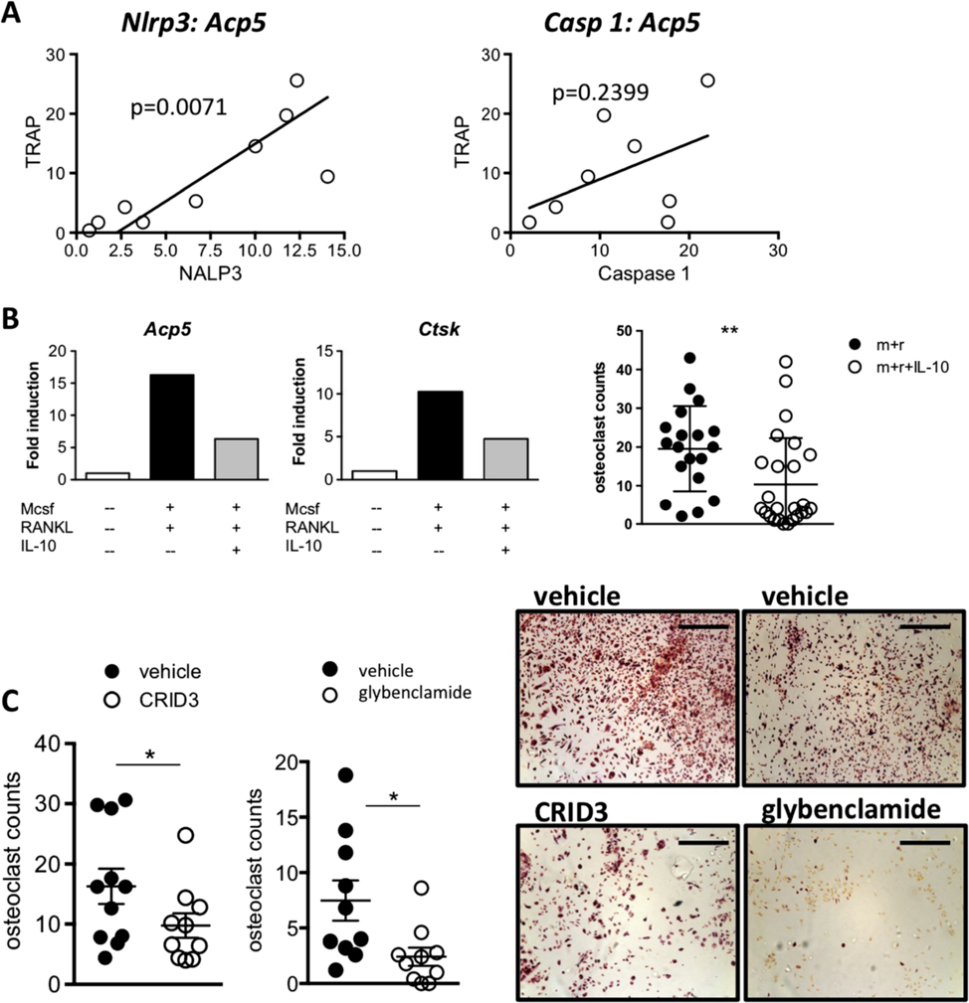
**IL-10 modulates inflammasome-driven osteoclastogenesis** ***ex vivo*** **and during antigen-induced arthritis (AIA). (A)** Correlation of *Nlrp3* and *Casp1* with *Acp5* in mRNA derived from the synovium of IL-10KO mice at day 28 post AIA. Values are relative to *18S* ribosomal RNA (n = 8-9). **(B)** Bone marrow cells from WT mice were cultured with MCSF in the presence (closed and open circles, left, black and grey bars, right) or absence (white bars, right) of RANKL and IL-10 (30 ng/ml) as indicated (open circles, left, grey bars, right). Values are derived from five independent fields of view from an experiment performed in triplicate. Representative pictures of cells treated with MCSF (left), MCSF and RANKL (middle) and MCSF, RANKL and IL-10 (right). Data was accumulated from two separate experiments. (Scale bar: 300 μm.) Expression of osteoclast markers; *Acp5* and C*tsk*, were determined by qPCR and compared against *18S* ribosomal RNA. **(C)** Bone marrow cells from IL-10KO mice were cultured RANKL and MCSF. Osteoclast formation was determined in the presence of vehicle alone (closed circles), 30 μM of CRID3 (open circles, left) or 25 μg/ml glibenclamide (open circles, right). Data is representative of three experiments performed in triplicate (^*^ *P* <0.05). Scale bar 300 μm. CRID3, cytokine release inhibitory drug-3; IL-10KO, interleukin-10-deficient; MCSF, macrophage colony-stimulating factor; qPCR, quantitative real-time PCR; RANKL, receptor activator of nuclear factor-κβ ligand; WT, wild-type.

We next sought to provide a link between arthritic bone erosions and the regulation of the inflammasome. Quantitative PCR revealed a positive correlation between *Acp5* and *Nlrp3* (r = 0.8173) and *Casp1* (r = 0.47) in samples from IL-10KO mice (Figure 5A). We therefore considered the involvement of IL-10 and the NLRP3 in an *in vitro* MCSF/ RANKL-driven osteoclast differentiation assay. Consistent with previous studies [13,15], IL-10 blocked osteoclast differentiation by approximately twofold (Figure 5B). Under these conditions, IL-10 inhibited *Acp5* and *Ctsk* (cathepsin-K, which contributes to osteoclast-mediated bone resorption) expression (Figure 5B). To assess the role of the inflammasome in this process, osteoclast formation was monitored in the present of cytokine release inhibitory drug-3 (CRID3) or glibenclamide. CRID3 inhibits NLRP3 and AIM2 by blocking ASC oligomers and the activation of caspase 1 [30], while glibenclamide is an ATP-sensitive potassium channel inhibitor that prevents maturation of caspase 1 and pro-IL-1β through NLRP3 [31]. Using myeloid progenitors from IL-10KO mice, addition of CRID3 or glibenclamide significantly reduced osteoclast numbers and blocked generation of TRAP-positive cells (Figure 5C). These data support a role for NLRP3 in governing bone turnover and erosion.

## Discussion

Innate sensing mechanisms were traditionally linked with the recognition of bacterial, fungal and viral infections. This viewpoint has, however, changed and many act as sensors of both endogenous and exogenous danger signals [32-34]. Members of the NLR family are intrinsic to caspase-activating complexes termed the inflammasome. These receptors recognize certain infectious pathogens, particles (for example, microcrystals), metabolic anomalies (for example, hyperglycaemia, ATP) and chemicals, and contribute to the pathology of various autoinflammatory diseases [32]. This has led to the novel application of drugs that target IL-1β (for example, anakinra, canakinumab and rilonacept) in conditions such as periodic fever syndromes, Still’s disease, Schnitzler’s syndrome, and gouty arthritis where conventional anti-inflammatory drugs fail to provide long-lasting relief [35]. Here, we show that IL-10 negatively regulates the expression of NLRP3 inflammasome components within the inflamed synovium of experimental arthritis and provide a link to degenerative bone erosion.

Several lines of evidence support an involvement of the inflammasome in inflammatory and degenerative arthritis. While NLRP3 is expressed in RA and OA synovium [36,37] it is difficult to comment on the functional significance of these findings as quantification of transcript levels provide minimal information on inflammasome activity. An involvement of the inflammasome in joint pathology is illustrated by studies of calcium crystals, which accumulate through biomechanical stress or altered mechanisms of calcification. Here, the ectopic deposition of hydroxyapatite crystals in synovial fluids from osteoarthritis patients is associated with disease progression [38]. Hydroxyapatite crystals activate the NLRP3 inflammasome to promote IL-1β and IL-18 release by lipopolysaccharide (LPS)-primed macrophages [24]. Studies in an air pouch model of synovitis also confirmed the activation of NLRP3/ASC/caspase 1 by hydroxyapatite crystals and supported a role in controlling neutrophil infiltration [24]. Analysis of synovial inflammation in AIA-challenged ASC-KO mice showed reduced disease severity [39]. This response was not, however, seen in NLRP3-KO and caspase1-KO mice, where both genotypes showed similar pathology to that observed in WT controls [39]. These results are consistent with our own, where we see similar disease pathology between the WT and IL-10KO mice at these early time points. We now show that at the later stages of inflammatory arthritis, in the absence of IL-10, joint inflammation results in a temporal increase in synovial IL-1β, which corresponds with enhanced pathology and co-localization to areas rich in F4/80-postive cells that cluster at sites of focal bone erosion. Such changes in IL-1β may reflect the capacity of IL-10 to inhibit TLR control of *Il1β* (pro-IL-1β) and the expression of NLRP3 inflammasome components. At this stage we are, however, unable to ascertain what inflammatory events (for example, TLR or cytokine-driven outcomes) regulate the induction of these inflammasome genes.

Our results implicate the involvement of the inflammasome in osteoclastogenesis, with IL-10 deficiency causing an increase in both synovial IL-1β and IL-33 expression, but not IL-18. Interleukin-1β is considered pro-osteoclastogenic and drives an imbalance in chondrocyte responses through regulation of prostanoids, nitric oxide and other free radicals, and the induction of degradative enzymes that impact collagen and proteoglycan turnover [40,41]. In contrast, IL-33 and IL-18 are considered anti-osteoclastogenic and protect against tumour necrosis factor alpha (TNFα)-mediated bone loss [42,43]. Thus, IL-33 often opposes the activities of IL-1β, which raises a question about the regulation of these cytokines by the NLRP3/caspase 1 system. While pro-IL-1β and pro-IL-18 are processed by caspase 1 into mature active forms, full-length IL-33 is already biologically active and is released as a consequence of cell damage [44]. Further processing by caspase 1 causes inactivation of IL-33 [44,45]. IL-33 is also modified by the activity of elastase and cathepsin G, which are secreted by infiltrating neutrophils and enhance IL-33 bioactivity [46]. Here, IL-33 acts as an endogenous danger signal (alarmin), which alerts innate immune cells to sites of infection or injury. Thus, the elevated expression of *Il33* in IL-10KO mice may simply reflect the overall increase in synovial inflammation. Moreover, the blockade of osteoclastogenesis by CRID3 and glibenclamide suggests a role for caspase 1 in bone turnover. Targeting the inflammasome may therefore benefit joint pathologies allied with IL-1β production, such as OA, Muckle-Wells syndrome (an autoinflammatory disorder linked with mutations in NLRP3) and gout [20,35,47,48]. Such an approach may provide an added benefit over traditional biologic interventions including anakinra, which in clinical trials of OA offered no improvement in symptoms [49]. To provide preclinical evidence to support this notion, attempts were made to treat AIA-challenged IL-10KO mice (i.a.) with either CRID3 or glibenclamide. However, administration (i.a.) of vehicle alone as a control promoted a robust inflammatory response, which prevented the direct investigation of this *in vivo*.

Our results indicate that IL-10 has the capacity to inhibit expression of NLRP3 and certain components of the inflammasome. Here, neutralization of IL-10 receptor signaling in WT mice enhanced synovial *Nlrp3* and *Casp1* expression and addition of endogenous IL-10 inhibited the formation of TRAP+ osteoclasts *in vitro*. Significantly, human trials with recombinant IL-10 showed no improvement in disease activity [50], while IL-10 responses in synovial macrophages from RA patients appear dysregulated [51]. Such alterations in IL-10 responsiveness results in a loss of its anti-inflammatory properties and a concomitant acquisition of interferon-gamma (IFNγ)-like activities [51].

## Conclusions

Our studies implicate a link between IL-10 and expression of the NLRP3 inflammasome within the inflamed synovium, and advocate a role for IL-10 and inflammasome activation in governing osteoclastogenesis and bone erosion. With advances in synovial histopathology taking centre stage in clinical practice [2], monitoring IL-10 or inflammasome activities within the inflamed joints of patients with early RA may prove valuable as a predictor of disease activity or bone erosions, and may help tailor the design of novel treatments for defined patient groups.

## Additional file

Additional file 1
**Oligonucleotide primer sequences for real-time PCR.** Oligonucleotide primer sequences for each of the inflammatory mediators measured in the study. All sequences are listed in the 5′-3′ direction.

## Abbreviations

AIA: antigen-induced arthritis
(M)CFA: (modified) Freud’s complete adjuvant
CRID3: cytokine release inhibitory drug-3
DKK-1: dickkopf-1
ELISA: enzyme-linked immunosorbent assay
i.a.: intra-articular
IL-10(KO): interleukin-10(-deficient)
i.p.: intraperitoneally
mBSA: methylated bovine serum albumin
MCSF: macrophage colony-stimulating factor
NLR: nucleotide-binding domain and leucine-rich repeat-containing receptors
OA: osteoarthritis
PBS: phosphate-buffered saline
qPCR: quantitative real-time PCR
RA: rheumatoid arthritis
RANKL: receptor activator of nuclear factor-κβ ligand
RLR: Rig-I-like receptors
s.c.: subcutaneous
TLR: Toll-like receptors
TRAP: tartrate-resistant alkaline phosphatase
WT: wild-type
