# Supplementary material for: Interleukin-10 regulates the inflammasome-driven augmentation of inflammatory arthritis and joint destruction
Source: Arthritis Res Ther. 2014 Aug 30;16(4):419. doi: 10.1186/s13075-014-0419-y (PMC4292830; doi:10.1186/s13075-014-0419-y)
Supplement: Additional file 1 — Oligonucleotide primer sequences for real-time PCR. Oligonucleotide primer sequences for each of the inflammatory mediators measured in the study. All sequences are listed in the 5′-3′ direction. [file 13075_2014_419_MOESM1_ESM.pdf]

Additional file 1- Oligonucleotide primers for real-time PCR
All sequences are listed in the 5'-3' direction

**Name**	**Sequence**
18S forward	GTAACCCGTTGAACCCCATT
18S reverse	CCATCCAATCGGTAGTAGCG
Aim 2 forward	ACAAAGTGCGAGGAAGGAGA
Aim 2 reverse	TCACTCCACACTTTTCATGTCA
ASC forward	GCAGCTGACTTCCTGGTCT
ASC reverse	ACGACTCCAGATAGTAGCTGACA
Acp5 forward	CAGCCCTTATTACCGTTTGC
Acp5 reverse	GAATTGCCACACAGCATCAC
CathepsinK forward	TGTCTGAGAACTATGGCTGTGG
CathepsinK reverse	ATACGGGTAACGTCTTCAGAG
Caspase 1 forward	ACGCCATGGCTGACAAGATCCTG
Caspase 1 reverse	GGTCCCGTGCCTTGTCCATAGC
Caspase 12 forward	AGGATGATGGACCTCAGAAG
Caspase 12 reverse	TCTCAGACTCCGACAGTTAG
IL-1b forward	CAACCAACAAGTGATATTCTCCAT
IL-1b reverse	GGGTGTGCCGTCTTTCATTA
IL-6 forward	ATGGATGCTACCAAACTGGAT
IL-6 reverse	TGAAGGACTCTGGCTTTGTCT
IL-18 forward	GCCATGTCAGAAGACTCTTGCGT
IL-18 reverse	GTACAGTGAAGTCGGCCAAAGTT
IL-33 forward	GATGGGAAGAAGGTGATGGGTG
IL-33 reverse	TTGTGAAGGACGAAGAAGGC
Nlrp1 forward	ATGTGGACCCAACCTTCAAA
Nlrp1 reverse	GTACGTGCTCCTGGAAAGGT
Nlrp3 forward	GCACCAACCGGAGCCTCACT
Nlrp3 reverse	CAGCGCCCCAACCACAGTCT
NOD1 forward	GGTTCTCTGCCTCTCCACTG
NOD1 reverse	ATCAGGGGAATCTGCTTGAC
NOD2 forward	GGTTCTCTGCCTCTCCACTG
NOD2 reverse	ATCAGGGGAATCTGCTTGAC
RIGI forward	CTGCCTCACTCTTCCTCCAG
RIGI reverse	TGGCTTCACAAAGTCCACAG
TNF forward	AGCCAGGAGGGAGAACAGA
TNF reverse	CAGTGAGTGAAAGGGACAGAAC
